# Estimating average inpatient and outpatient costs and childhood pneumonia and diarrhoea treatment costs in an urban health centre in Zambia

**DOI:** 10.1186/1478-7547-7-16

**Published:** 2009-10-21

**Authors:** Lumbwe Chola, Bjarne Robberstad

**Affiliations:** 1 Central Statistical Office, Box 31908, Lusaka, Zambia; 2 Centre for International Health, University of Bergen, Box 7804, N-5020 Bergen, Norway

## Abstract

**Background:**

Millions of children die every year in developing countries, from preventable diseases such as pneumonia and diarrhoea, owing to low levels of investment in child health. Investment efforts are hampered by a general lack of adequate information that is necessary for priority setting in this sector. This paper measures the health system costs of providing inpatient and outpatient services, and also the costs associated with treating pneumonia and diarrhoea in under-five children at a health centre in Zambia.

**Methods:**

Annual economic and financial cost data were collected in 2005-2006. Data were summarized in a Microsoft excel spreadsheet to obtain total department costs and average disease treatment costs.

**Results:**

The total annual cost of operating the health centre was US$1,731,661 of which US$1 284 306 and US$447,355 were patient care and overhead departments costs, respectively. The average cost of providing out-patient services was US$3 per visit, while the cost of in-patient treatment was US$18 per bed day. The cost of providing dental services was highest at US$20 per visit, and the cost of VCT services was lowest, with US$1 per visit. The cost per out-patient visit for under-five pneumonia was US$48, while the cost per bed day was US$215. The cost per outpatient visit attributed to under-five diarrhoea was US$26, and the cost per bed day was US$78.

**Conclusion:**

In the face of insufficient data, a cost analysis exercise is a difficult but feasible undertaking. The study findings are useful and applicable in similar settings, and can be used in cost effectiveness analyses of health interventions.

## Background

The challenge to meet the millennium development goal of reducing under-five mortality with two thirds by 2015 has prompted the need for increased investment in child health in low income countries, where about 4.8 million children die every year from preventable diseases [1]. More than 70 per cent of these child deaths are attributable to diseases such as diarrhoea, malaria and pneumonia [2]. Pneumonia and diarrhoea together account for more child deaths than any other single causes of death [3].

Morbidity and mortality due to these and other diseases impose a huge economic burden on individuals, families and on society at large. This economic burden is more acute in developing countries, where the opportunity costs of resources are very high. This necessitates the need for priority setting in the allocation of resources.

However, priority setting in most developing countries is hampered by lack of sufficient information [4]. Little is known about the costs and effects of various intervention strategies employed to combat diseases. Even where they might be known, data tend to be poorly documented and financial rather than economic costs are collected.

A literature search was conducted between June 2006 and February 2007, for English language articles on healthcare costs and the costs of pneumonia and diarrhoea in the following databases: MEDLINE (January 1966 to date), Cochrane Controlled Trials Register (CENTRAL), the Cochrane economic evaluation database (NHS EED), EMBASE-Medicine (January 1985 to date) and the ISI Web of Science. The search did not yield any studies undertaken in Zambia to measure health care costs or treatment costs of pneumonia and diarrhoea in children. Limited data are also available on the costs of general healthcare services [5,6] and the economic burden of diseases such as pneumonia and diarrhoea in developing countries [7-11].

In an effort to document information that is potentially beneficial to priority setting in the health sector, we set out to measure the health system costs of providing inpatient and outpatient healthcare services to an urban population in Zambia. We demonstrated how our findings can be used to measure disease specific costs by calculating average treatment costs of pneumonia and diarrhoea in under-five children. This was also done in order to justify the adoption of cost-effective disease prevention measures. Research has shown the efficacy of rotavirus and pneumococcal vaccines in preventing diarrhoea and pneumonia in children [12,13], and our study findings can be integrated into cost effectiveness studies for these vaccines.

## Methods

### Study area

Lusaka district, in which the capital of Zambia is situated, has a population of about 2 million people. Healthcare for the district is provided by the central Government and private for profit institutions, which are mainly situated in urban areas. The public health institutions are classified into health posts, health centres, district referral centres, general hospitals and central hospitals. Health posts are intended to cater for a population of about 500 to 100 households, while health centres serve a population of about 50,000 people in urban areas and 10,000 people in rural areas. District referral centres, general and central hospitals are meant to cater for populations of 200,000; 800,000 and above 800,000 respectively. Lusaka district has 8 health posts, 22 health centres and 2 central hospitals.

We undertook the costing study at Kanyama health centre, in Kanyama Township in the capital, Lusaka. The health centre is a public institution funded by the Ministry of Health (MOH), under the Lusaka District Health Management Team (DHMT). Its catchment area includes all the households in Kanyama Township and some other households in surrounding areas. The centre provides both outpatient and inpatient services, and has about 700 visits daily, and a total of 41 beds.

Kanyama Township had an estimated population of over 130,000 in 2006 [14]. Approximately 20% of the population were children below the age of 5 years. The Township has widespread poverty and unemployment and is characterised by poor sanitation and infrastructure. Urban poverty is quite high in Lusaka, with about 53% of all households living in absolute poverty [15]. Unemployment is also high, and was estimated at 28% in 2005 [16]. Poverty mostly affects women and children, who constitute over 70% of the poor population.

### Perspective

The study was undertaken from the health provider perspective, and as such, only costs borne by the health centre were taken into consideration. We did not include the costs to households and social costs such as quality of life reductions caused by disease.

### Costs

Data collection was guided by the *Cost Analysis in Primary Health Care: A Manual for Programme Managers *[17]. Cost and outcome indicators were collected for the period August 2005 to August 2006. Since the health centre is a government institution, all its resources were provided by the government, or by other agencies such as non-governmental organisations and the donor community through the government. Therefore, only information about the quantities of resources used in the provision of care and on disease outcomes was obtained from the health centre records. Most of the data used in this study was collected from the MOH Health Management Information System (HMIS). This is an information system that is part of the monitoring and evaluation framework of the health sector in Zambia [18]. The HMIS is a routine data collection system designed to provide information on the performance of the health system. Data such as number of visits, bed days, disease cases, and laboratory tests is collected on a monthly basis in the HMIS, a system which has been used for over a decade at Kanyama health centre.

Data on the prices and quantities of drugs and other recurrent medical supplies provided to the health centre were obtained from the DHMT pharmacy, which supplies all such items to government health centres in Lusaka District. It has a well developed system of delivery with updated documentation of all transactions to health centres. We were able to access these records to obtain updated information on supplies to Kanyama health centre, and accurate prices of most of the items provided. The prevailing market price was used as proxy for items whose prices were unavailable in the records of both the health centre and the DHMT. All costs were adjusted to 2006 prices using the 2006 Gross Domestic Products (GDP) deflator, valued at K1,175 [19]. Items were valued in Zambian Kwacha (K) and converted to United States Dollars (US$) at the average exchange rate in the study period, of about K3,600 to US$1 [19].

Costs were classified as either economic or financial. Economic costs reflected the opportunity cost of resource use, while financial costs considered only expenditures incurred in the purchase of items. Costs were further classified as capital or recurrent. Capital costs included items such as buildings, equipment and vehicles, who's useful life was more than one year. Recurrent costs included items such as personnel and supplies, which could be replaced within a year. Items such as cutlery, buckets and garden implements, which could last for more than a year but cost less than US$100 were regarded as recurrent costs.

A physical count of all capital items such as furniture and equipment was undertaken to ascertain the exact number and condition. Only items that were functional were included for costing. The floor areas and costs of buildings were obtained from the Buildings Department at the Ministry of Works and Supply, which is the custodian of all information on government buildings. Where such costs were not available, the floor space of the buildings was measured physically and an estimated value was attached by a Quantity Surveyor at the Department.

Capital costs were annuitized to take into account the fact that such resources are bought in one year, but their useful life spans over several years [20]. The annual financial cost of capital items was calculated using a straight line depreciation method, where an item's total cost was divided by the length of its useful life years. The economic cost of capital items was calculated using an interest rate of 6%, as recommended in literature [21]. All five buildings of the health centre were assumed to have a useful life of 30 years, while the useful life of equipment varied from about 2 to 20 years. The useful life years for all capital items, except buildings, were obtained from the costs and prices used in the WHO Choosing Interventions that are Cost Effective (WHO-CHOICE) analysis [22].

Data were, however, sometimes not readily available and had to be estimated using proxies. For example, we did not have access to the original records of some capital and recurrent outlays, which made it difficult to obtain their original purchase prices. We therefore, made estimates which could have been over or under the true value. This was the case also for utilities, for which we did not find any records on bills paid towards water and electricity. We decided to use fixed monthly charges of US$2.78 for water [23] and US$6.50 for electricity [24] based on the prevailing rates in the study area. The utilities cost was therefore only considered as an economic and not a financial cost.

In some cases, the records were available but not in order, and the researcher could not sort them due to inadequate time and resources. It was not possible, for example, to allocate some medical supplies received by the health centre's pharmacy to the various wards or departments, as usage of these items was not specified by ward. It was thus decided to treat all such items as overhead costs, to be allocated to each of the wards according to an estimated allocation factor. The supplies that were 'ward or department specific', e.g. family planning drugs and some antibiotics were allocated directly to the necessary wards or departments.

Further, it was not immediately possible to allocate certain items, such as staff time to certain departments, as their usage between departments was not clearly defined. The same personnel who tended to the paediatric ward also serviced the male and female wards. As a result, it was not easy to distinguish to which ward/department some of the staff time belonged. It was thus decided to allocate time equally to those departments that were serviced by the same personnel, as there was no clearly defined pattern of work. The same was done for equipment, where percent usage was divided equally between wards.

### Organizational structure and production function

**Figure 1:**
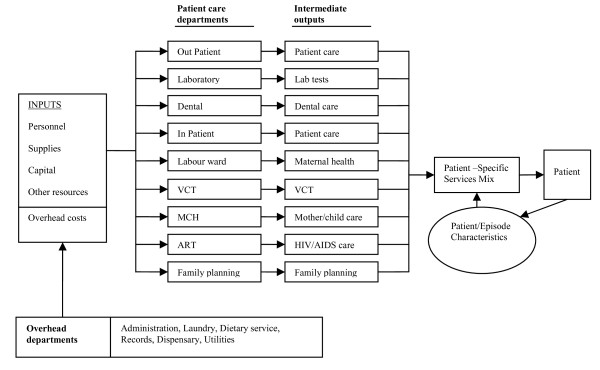
**Kanyama health centre production function model**. Figure 1 shows the health centre's organizational structure and service production model, which illustrates how the health centre used a number of inputs (such as personnel and equipment), to produce services (such as lab tests and patient care) that patients received. These services are produced by the patient care departments (such as the out-patient, laboratory and in-patient). This is done by way of combining the available inputs. The patient specific mix of intermediate products received is driven by a number of patient characteristics that are unique to each visit. These characteristics include demographic (e.g. sex, age) and clinical (e.g. diagnoses) information [25]. Patient care departments also use services from other departments that do not directly lead to the production of intermediate outputs. These are the overhead departments, such as administration, dispensary and laundry. The services produced by these departments are indirect costs incurred by each patient care department.

Figure 1 shows the health centre's organizational structure and service production model, which illustrates how the health centre used a number of inputs (such as personnel and equipment), to produce services (such as lab tests and patient care) that patients received [25].

The health centre's departments were classified into 9 patient care and 6 overhead departments. The patient care departments included all units or wards where patients directly received treatment, such as the inpatient and outpatient wards and the laboratory. The overhead departments were those that serviced the patient care departments and whose activities were not immediately identified with disease treatment [17,20]. These included units such as the laundry, kitchen, pharmacy and general administration.

In each department, costs were categorised as follows: Personnel cost, including salaries, benefits and allowances. Capital costs, including buildings, vehicles, furniture and equipment such as x-ray machines. Supplies, including cleaning supplies, medical and surgical supplies. Maintenance, for buildings, vehicles and general repairs. For inpatient costs, we included beds, inpatient maintenance and supplies and length of stay for each disease.

The costs were further divided into patient care costs, which were costs incurred in the patient care departments, and overhead costs, incurred in the overhead departments. Overhead departments' costs were shared by all the patient care units. These costs were allocated to each patient care department according to the estimated proportion of number of visits, bed days, personnel or floor space.

The intermediate outputs were the services provided by each patient care department. The units of analysis used to measure the output were bed days for the inpatient department and visits for the outpatient department. In the laboratory, the units of measurement were the total number of lab tests conducted in the study period. In the labour/maternity ward, the units used were visits instead of bed days. This is because the majority of the women spent a day or less in this ward before being discharged. The total number of visits and admissions included both persons attending the health centre for the first time (new attendees) and re-attendees.

### Outputs and average costs

Total costs were divided by total outputs to obtain average costs. The average cost of inpatient care, for example, was obtained by dividing the total cost in the paediatric ward by the total number of bed days in the reference period. Average costs of service provision are presented for all the patient care departments except Anti Retroviral Treatment (ART), for which information on usage was not available.

### Pneumonia and diarrhoea treatment costs in under-five children

To decide on the introduction of pneumococcal and rotavirus vaccines, the economic burden of pneumonia and diarrhoea is needed for inclusion in cost-effectiveness studies. Our result can help estimate these costs. To do this, we measured the average treatment costs of pneumonia and diarrhoea, using total under-five pneumonia and diarrhoea visits and bed days recorded in the health centre's records, and the total outpatient and in-patient costs for treating these diseases. All resources related to the treatment of childhood pneumonia, including personnel, buildings, medical supplies, laboratory equipment and utilities were costed. Unit costs for each disease were calculated by dividing the total outpatient and inpatient costs by the corresponding visits or bed days for the condition. The average healthcare costs provided for both pneumonia and diarrhoea thus include the outpatient (cost per visit) and inpatient (cost per bed day) costs.

## Results

### Costs of patient care departments

The annual costs incurred in the patient care departments are presented in table 1. The total direct costs amounted to US$1,284,305 with US$1,195,129 attributed to recurrent costs and US$89,176 to capital costs. Capital costs contributed about 7% of total costs.

**Table 1:** Annual patient care departments costs, 2006 US$

	**Recurrent costs**		**Annualised Capital costs**		**Total costs**	
	**US$**	**%**	**US$**	**%**	**US$**	**%**
Out-Patient Department	418,079	35	8,768	10	426,847	33
In-patient department	164,914	14	25,331	28	190,245	15
Laboratory Department	32,444	3	7,269	8	39,713	3
Dental Department	19,949	2	2,389	3	22,338	2
Labour/postnatal ward	80,297	7	14,071	16	94,368	7
VCT Ward	8,330	1	1,329	1	9,659	1
Mother and child health department	103,515	9	11,903	13	115,418	9
ART Department	339,570	28	17,513	20	357,083	28
Family planning department	28,031	2	603	1	28,634	2
						
**Total**	**1,195,129**	**100**	**89,176**	**100**	**1,284,305**	**100**

The outpatient ward had the highest total cost at US$426,847 (33%), while the Voluntary Counselling and Testing Ward (VCT) ward had the lowest at US$9,659 (1%). The outpatient ward also had the highest recurrent cost (35%), while the inpatient ward had the highest capital cost (28%).

### Kanyama health centre output

Table 2 presents the output recorded during the study period. Kanyama health centre had an equivalent number of 150 staff in full time employment. It recorded approximately 208,964 visits, with 2,894 admissions and 10,633 bed days. A total number of 49,628 visits and 1,309 admissions were attributed to pneumonia and diarrhoea. There were a total number of 93,717 visits and 1,424 admissions made by children under the age of 5 years. About 16,511 and 8,925 visits; and 607 and 221 admissions made by under-five children were attributed to diarrhoea and pneumonia, respectively.

**Table 2:** Kanyama health centre output

**Output**	
**Capacity/use**	
Total number of staff	150
Total number of visits	208,964
Total number of admissions	2,894
Total number of bed-days	10,633
Total visits <5 years	93,717
Total admissions<5 years	1,424
	
**Pneumonia and diarrhoea capacity/use**	
Total visits (Pneumonia/diarrhoea)	49,628
Total admissions (Pneumonia/diarrhoea)	1,309
Total <5 years visits (pneumonia)	8,925
Total <5 years visits (diarrhoea)	16,511
Total <5 years admissions (pneumonia)	221
Total <5 years admissions (diarrhoea)	607
	
**Adjustment**	
Average length of stay	4
Bed occupancy rate	82%
Bed turn over	71%
Visits/personnel	14

There were an estimated 14 outpatient visits per day per medical personnel for the entire study period. The health centre's bed occupancy rate, defined as the bed days per number of beds was 82%; while the bed turnover, defined as the number of admissions per number of beds was 71%. The average length of stay was 4 days.

Table 3 shows the outputs in all patient care departments. The out-patient department had the most visits at 128,240 and the dental had the least with 1,091. The total number of bed days recorded in the inpatient department was 10,633. There were 13,370 lab tests undertaken during the study period.

**Table 3:** Kanyama health centre output by department/ward, 2006 US$

**Department/ward**	**Unit**	**Number**
Out-Patient Department	Visits	128,240
In-patient Department	Bed days	10,633
Laboratory Department	Tests	13,370
Dental Department	Visits	1,091
Labour/postnatal ward	Visits	15,382
VCT Ward	Visits	6,500
Mother and child health department	Visits	53,501
ART Department	Visits	-
Family planning department	Visits	4,250

### Average costs

The average costs per unit of service in all the departments are given in table 4. The cost per visit in the outpatient department was US$3, while the cost per dental visit was US$20. The VCT ward recorded the lowest cost per visit at US$1. The inpatient department recorded a cost per bed day of US$18.

**Table 4:** Average costs per visit/bed day (all departments and wards), 2006 US$

**Department/ward**	**Unit**	**US$ per visit/bed day**
Out-Patient Department	Visits	3
In-patient Department	Bed days	18
Laboratory Department	Tests	3
Dental Department	Visits	20
Labour/postnatal ward	Visits	6
VCT Ward	Visits	1
Mother and child health department	Visits	2
ART Department	Visits	-
Family planning department	Visits	7

### Pneumonia and diarrhoea treatment costs in under-five children

Table 5 shows the average cost of pneumonia and diarrhoea treatment in under-five children. The average cost of diarrhoea treatment in children under the age of 5 years was US$26 per visit and US$78 per bed day. The cost of pneumonia was US$48 per visit and US$215 per bed day.

**Table 5:** Average costs per visit/bed day (under-five pneumonia and diarrhoea), 2006 US$

**Department/ward**	**US$ per visit/bed day**
**Diarrhoea**	
Out-Patient Department (Under 5)	26
Paediatric Ward	78

**Pneumonia**	
Out-Patient Department (Under 5)	48
Paediatric Ward	215

## Discussion

This paper provides information on the average outpatient and inpatient treatment costs in an urban health centre in Zambia, and also measures the average cost of pneumonia and diarrhoea treatment in children under the age of 5 years. The average costs incurred in the patient care departments ranged from US$1 to US$20, with the highest cost observed in the dental department. Except for dental services, all out-patient services costs were relatively much lower than in-patient costs. This is reflective of the severity of inpatient cases, which require relatively more care and consequently more resources to manage. Additional costs such as lodging facilities and meals tend to increase admission costs [20]. The high costs of inpatient treatment necessitate the need for preventive care interventions, which could lead to a reduction in the number of disease cases, and subsequently a reduction in health care costs.

We estimated the total annual cost of operating the health centre at US$1,284,305. It was however not possible to isolate the government budget for Kanyama health centre and compare it with our result. Most of the budget components were financed through various departments, and aggregated together with information from other clinics and health centres. The salaries, for example were paid through MOH and the health centre's supplies and equipment were provided by DHMT.

The government per capita expenditure on health was US$17 in 2005 [26]. Taking this in the context of Kanyama health centre and its catchment population area of 134,000, the ratio of health centre costs to population is just about US$10 per capita. Whether this investment is adequate for the good health of this population is uncertain, and is beyond the scope of this paper. It is interesting to note though, that the average cost of in-patient services at US$18 is higher than the per capita expenditure on health. This highlights the need for increased expenditure to the health sector, and again, the need for preventive measures to free up much required resources.

The annual per capita cost of providing healthcare calculated in this paper is lower than the cost estimated by the World Bank [27], but is comparable to an estimate made by a study similar to ours undertaken in Zimbabwe [5]. The World Bank study estimated annual per capita costs in the range of US$13 to US$16 for Africa, while the Zimbabwean study reported a cost of US$10 per capita. The range of outpatient treatment costs we report US$1 to US$20, also compares well with costs of curative contacts and preventive care recorded in the Zimbabwe study (US$2 - US$23). It should be noted however, that while the methodology of the Zimbabwe study is similar to ours, data collection was done at district level involving several hospitals and health centres, while ours was undertaken at only one health centre. Therefore, the treatment options in our study, including drugs are likely to be relatively inexpensive.

In comparison to a study undertaken in Kenya, which recently reported costs of in-patient pneumonia treatment in children, ranging from about US$46 to US$172 [8], our estimate of US$215 is much higher. A similar study in Pakistan reported average treatment costs of US$71 for pneumonia and US$236 for severe pneumonia, a distinction which we did not make in our study [9]. Fuchs et al [11] reported that the Brazilian government spent US$ 135 per pneumonia admission in 2003. This figure, however, also included children between the ages 5-14 years. Though the objective of this study was to highlight the burden of disease, the author did not point to the source of this cost information and the methodology used to arrive at this cost. Taking a provider perspective, Constenla found that the costs of treating pneumonia varied greatly in some Latin American countries, ranging between US$372 to US$3,483 per child [7]. In the case of diarrhoea, the WHO [10] reported an average cost of diarrhoea in Mexico of $190, $37 in India and $66 in The Philippines, from reviews of studies undertaken by researchers in the respective countries.

A limitation of this study is that it did not measure the supervisory costs incurred by the Ministry of Health, and the costs of support incurred by non-governmental organisations and the donor community, thereby underestimating the true health systems cost of service provision to Kanyama Township. Furthermore, our exclusion of family costs incurred in accessing healthcare does not give a true reflection of the burden of disease and healthcare. It is necessary for social planning to also have an insight into the cost structures of individuals, households and society at large. Future research should consider both the direct and indirect costs of treatment, and the long term effects of quality of life reductions due to illness. This perspective is more informative to policy makers as it helps them plan for a wider section of the community.

The paper, however, fills a large gap in the literature on the cost of healthcare provision in Zambia, particularly on the cost of childhood pneumonia and diarrhoea to the health system. It underscores the challenge of undertaking a cost analysis in the face of unavailability of reliable, consistent and accurate data, which is the case in many developing countries [17,20]. In Zambia, as in many other sub-Saharan African countries, cost data in the health and other sectors is not part of routine statistical data collection activities, which makes it difficult to immediately obtain reliable statistical information.

The study provides information that is potentially useful for planning in the area of child health. It documents the procedures for undertaking cost analyses of healthcare services which can be used in similar settings. Our experiences in this study could be a guide for others as far as highlighting what is expected in the course of undertaking such work.

This study was undertaken in a particular institutional and epidemiological setting. Although generalization must be made with great caution, the study can still be useful in similar Zambian settings since the costs of large items such as staff, equipment and drugs are fairly standard. The costs of medication, treatment and other facility costs may also be generally comparable.

## Competing interests

The authors declare that they have no competing interests.

## Authors' contributions

Both authors participated in the design and planning of the study. Field work was conducted by LC; the analysis and write-up was carried out mainly by LC. Both authors read and approved the final manuscript.
